# Can Depression be Diagnosed by Response to Mother's Face? A Personalized Attachment-Based Paradigm for Diagnostic fMRI

**DOI:** 10.1371/journal.pone.0027253

**Published:** 2011-12-13

**Authors:** Xian Zhang, Zimri S. Yaseen, Igor I. Galynker, Joy Hirsch, Arnold Winston

**Affiliations:** 1 Department of Radiology, Department of Psychology, Hatch Research Center, Columbia University, Neurological Institute, New York, New York, United States of America; 2 Department of Psychiatry and Behavioral Sciences, Beth Israel Medical Center, New York, New York, United States of America; 3 Department of Psychiatry and Behavioral Sciences, Albert Einstein School of Medicine, Beth Israel Medical Center, New York, New York, United States of America; Institute of Psychiatry at the Federal University of Rio de Janeiro, Brazil

## Abstract

**Objective:**

Objective measurement of depression remains elusive. Depression has been associated with insecure attachment, and both have been associated with changes in brain reactivity in response to viewing standard emotional and neutral faces. In this study, we developed a method to calculate predicted scores for the Beck Depression Inventory II (BDI-II) using personalized stimuli: fMRI imaging of subjects viewing pictures of their own mothers.

**Methods:**

28 female subjects ages 18–30 (14 healthy controls and 14 unipolar depressed diagnosed by MINI psychiatric interview) were scored on the Beck Depression Inventory II (BDI-II) and the Adult Attachment Interview (AAI) coherence of mind scale of global attachment security. Subjects viewed pictures of Mother (M), Friend (F) and Stranger (S), during functional magnetic resonance imaging (fMRI). Using a principal component regression method (PCR), a predicted Beck Depression Inventory II (BDI-II) score was obtained from activity patterns in the paracingulate gyrus (Brodmann area 32) and compared to clinical diagnosis and the measured BDI-II score. The same procedure was performed for AAI coherence of mind scores.

**Results:**

Activity patterns in BA-32 identified depressed subjects. The categorical agreement between the derived BDI-II score (using the standard clinical cut-score of 14 on the BDI-II) and depression diagnosis by MINI psychiatric interview was 89%, with sensitivity 85.7% and specificity 92.8%. Predicted and measured BDI-II scores had a correlation of 0.55. Prediction of attachment security was not statistically significant.

**Conclusions:**

Brain activity in response to viewing one's mother may be diagnostic of depression. Functional magnetic resonance imaging using personalized paradigms has the potential to provide objective assessments, even when behavioral measures are not informative. Further, fMRI based diagnostic algorithms may enhance our understanding of the neural mechanisms of depression by identifying distinctive neural features of the illness.

## Introduction

Though depression is a leading cause of morbidity and mortality worldwide, our understanding remains incomplete [1]. Previous research has linked depression to functional and structural changes in the brain [2], [3], [4], [5], [6], [7], [8], and emerging work has used standard emotional faces paradigms during functional magnetic resonance imaging (fMRI) in combination with non-linear modeling techniques to diagnose depression [9]. Though powerful, non-linear modeling techniques may have limited generalizability and interpretability because of potential for over-fitting the data and model complexity [10]. On the other hand, studies of the neural correlates of depression generally report significant group differences in multiple regions of brain activity for depressed versus control subjects. [11] These differences may be more readily interpretable, but are not robustly sensitive or specific. Thus, a methodology that could detect brain activity patterns that were readily interpretable as well as sensitive and specific in regard to depression severity might enhance our understanding of the neural basis of depression. Further, such methods could ultimately lead to cost-effective tools to distinguish unipolar depression from similar disorders [12].

Growing evidence links failures in early attachment experience to depression [13], [14], [15], [16] Moreover, the linkage between attachment disruptions in childhood and emotional dysregulation and depression may be mediated via effects on the hypothalamic-pituitary-adrenal axis and the oxytocin-vasopressin system which modulates it. [17], [18], [19]. Because of the significant impact of attachment on depression, we hypothesized that brain activity in a personalized attachment related task, using images of one's own mother – the person who shapes attachment – could effectively detect depression and insecure attachment. In a previous study of depression, a personalized activation paradigm proved to be more effective than a standard one [20]. Thus, we hypothesized that a linear model applied to such a personalized attachment-related paradigm could operate robustly and provide interpretable results. Whether depression or attachment security scores are linearly related to brain activity levels is an empirical question and is tested in this study.

To test these hypotheses we developed predictive models of depression severity and attachment security based on a regression analysis between fMRI data and established depression (the Beck Depression Inventory-II, henceforth BDI-II) and attachment security (the Adult Attachment Interview coherence of mind score, henceforth AAI) ratings.

Our approach is motivated by the limitations involved in current approaches to fMRI-based diagnosis of depression. Using fMRI to diagnose a psychiatric disorder has been challenging due to both the relatively low signal-to-noise ratio of fMRI images and high variability across subjects. In general, improving fMRI-based psychiatric diagnosis should further our understanding of the neural mechanisms of psychopathology. Such improvement depends, however, on the discovery of more effective experimental paradigms, as well as technical advances in the processing of the high-dimensional data that results.

Some widely used techniques include partial least squares regression (PLS) [21], multi-voxel pattern analysis (MVPA), and support vector classification (SVC) [22], [23]. The essence of those approaches is multivariate analysis (MVA), i.e. using a data-driven approach to find the best combination of the multiple components of fMRI input that either maximizes the prediction accuracy or minimizes the regression error. Thus, although very high accuracies, such as 95% correct for diagnosing major depressive disorder (MDD) [22], have been reported, these accuracies were obtained for moderately to severely depressed subjects with low variability in Hamilton Rating Scale for Depression (HAM-D) scores. With higher variability in depression ratings and subject characteristics Hahn, et al., were able to predict with 83% accuracy [9]. Thus, finding ways to overcome major challenges inherent in multivariate analysis may further improve diagnostic accuracy of fMRI in depression.

Because the dimension of whole-brain fMRI data – approximately 2×10^5^ 2 mm^3^-voxels – is much greater than the number of subjects involved in any fMRI study, and the fMRI data are often highly variable, without appropriate cross-validation, MVA solutions may not generalize beyond the data set on which they were based. Dimension reduction is therefore a part of any MVA approach. For PLS, the data is converted into a few latent variables that are best correlated with the dependent variables, For SVC, only a handful of features are selected to represent the fMRI data. However, the effective dimension of the classifier is close to the product between number features and number of informative samples. These approaches are data-driven rather than signal-to-noise driven in that latent variable or feature selection is determined by the performance of the model as a classifier rather than by the signal-to-noise ratio of the voxels. The complexity of either the latent variables in PLS or the classifier of SVC, makes the specific models generated by these approaches difficult to interpret clinically. In most cases, they are large matrices that often tend to defy clinical interpretation, and it may be very difficult to describe how a prediction is made in a manner that provides insight into the neural mechanisms of a psychiatric disorder.

In this study, we adopted an approach based on principal component regression (PCR) – a combination of multiple linear regression (MLR) and principal component analysis (PCA). Although it is also a form of MVA, we have sought to minimize the data-driven component of the approach in limiting the input dimension, and limiting the options for nonlinear relationships. In order to avoid over-fitting and improve model interpretability, we identify a small set of voxels closely linked to the clinical data. Furthermore, the PCs of fMRI used for prediction were determined based on the variance of fMRI BOLD signal itself rather than being selected for their performance (in terms of *a posteriori* classification accuracy of the model).

We combined this approach to data analysis with a personalized paradigm (viewing one's own mother, friend, and others). Therefore, our results lend themselves to straightforward interpretation which may provide insight into the mechanisms of depression by identifying patterns of brain activity linearly related to the clinical measure.

In summary, we aimed to derive BDI-II and AAI from brain activity associated with viewing of attachment figures, and in particular, mothers. Our goal is to develop an objective measure identifying the patterns of brain activity sensitive and specific for depression as well as a better understanding of the relationship between attachment security and depression. Ultimately, fMRI based diagnosis could be both cost-effective and valuable in diagnosing unipolar depression in the presence of conditions whose clinical presentation may mimic it (bipolar disorder, schizophrenia prodrome), but whose treatment differs radically [12].

## Materials and Methods

### 1.1 Subjects and psychometric data

Twenty-eight right-handed women aged 19–31 years, fourteen depressed, based on *the Mini-International Neuropsychiatric Interview (MINI)*, and fourteen without history of psychiatric disorder were recruited by advertisement and assessed. The study was approved by the Beth Israel Medical Center Institutional Review Board. All participants signed the informed consent prior to participation.

Inclusion criteria were:

1. Right-handed females, age 18–30 years at time of recruitment
2. Able to understand and sign the informed consent
3. Raised (birth to at least 14 years old) in a household with their biological mother
4. Mother living and able to provide recent photographs
5. Fluency in English, and normal or corrected to normal visual acuity
6. For depressed subjects, current depression defined by the Mini-International Neuropsychiatric Interview

Exclusion criteria were:

1. Current and lifetime substance abuse
2. History of head trauma or mental retardation
3. History of Schizophrenia, Schizoaffective Disorder, Obsessive-Compulsive Disorder, or serious medical illness
4. Use of psychotropic medications: current or past year (depressed); lifetime (controls)
5. Acute suicidality

Of the 47 depressed women who responded to our advertisement for depressed subjects, 21 passed the telephone screen for inclusion/exclusion criteria and were interviewed on site. Of these, 14 depressed subjects met inclusion criteria and successfully underwent the complete fMRI scanning procedure. Their BDI-II scores ranged from 11–53, encompassing the range from mild to severe depression.

Of the 64 women who responded to our advertisement for control subjects, 36 passed the telephone screen and were interviewed on site. Fifteen were determined to be without current or lifetime psychiatric disorder, met inclusion/exclusion criteria, and underwent the fMRI scanning procedure. One subject was excluded due to excessive movement (>5 mm) in scanning.

### 1.2 Instruments and Subject evaluations

*The Mini-International Neuropsychiatric Interview (MINI)*, a short structured diagnostic interview for DSM-IV and ICD-10 psychiatric disorders, was used to establish subjects' clinical diagnosis of depression [24].

*The Beck Depression Inventory II (BDI-II)* was used to assess depression. Scores of 14–19 are considered mild, 20–28 moderate, and 29–63 severe depression [25].

Attachment security was assessed with the *Adult Attachment Interview (AAI)*. The AAI is a structured semi-clinical interview focusing upon early attachment experiences and their effects. From these interviews the Coherence of Mind index is derived as a measure of attachment security with values ranging from 1 to 9. Scores (henceforth referred to as ‘AAI scores’) 6–9 indicate secure attachment, scores 1–3 indicate insecure attachment, and scores 4–5 are indeterminate [26].

All MINI evaluations were conducted in the research office at the Beth Israel Medical Center 1–4 weeks prior to the scan. AAI and BDI-II measures were administered on the morning of the scan at the Hatch Imaging Center at Columbia Presbyterian Medical Center.

### 2.1 fMRI acquisition, experimental paradigm, and first level analysis

Scanning was performed on a Philips Intera 3T scanner using a Philips SENSE head coil (gradient echo EPI, TR/TE = 2 s/25 ms, 77° flip angle. Voxel size was 2×2×3 mm). Functional imaging data were preprocessed and analyzed with FSL (FMRIB Software Library) [27]. Motion correction parameters and global average of the BOLD for white matter were entered as covariates to control for movement and global BOLD signal fluctuation. Images were smoothed with a 9-mm FWHM Gaussian kernel.

The subjects randomly viewed images of their mother (early attachment), a female friend (late attachment), and female strangers during four 12.6-minute fMRI scanning runs. Each run consisted of 3 blocks. In a given block subjects performed one of three tasks: Subjects were instructed to respond according to “How much can you relate to this picture?” (Relatedness task), “How pleasant do you feel when you look at this picture?” (Valence task), and “Press any button when you see the picture” (Passive task) via a 1–4 button press (1–2 = negative to neutral, 3–4 = positive-very positive). To maximize power all three blocks were pooled.

Thus, there were three event related models: viewing images of Mother (M), Friend (F), and Strangers (S), respectively. The models were convolved with the canonical hemodynamic response function. Three contrasts, Mother vs. Friend (M–F), Mother vs. Strangers (M–S) and Friend vs. Strangers (F–S) were averaged using fixed effects analysis and were the fMRI input for subsequent PCR analysis.

### 2.2 The principal component regression (PCR) method

We used a leave-one-out approach to derive our predictive model. To predict BDI-II and AAI scores for each subject, data from the other 27 subjects was used to generate the model – a linear transformation mapping fMRI data onto the psychometric data. This map was then applied to the test subject's fMRI data to produce the model's prediction of the test subject's BDI-II and AAI scores. To derive this map, the dimension of the fMRI data was first reduced in two steps.

First, the region of interest (ROI) was determined with a general linear model (GLM) analysis using the standard mixed effect group analysis provided by FSL [28]. The contrast images of (M–F), (M–S), and (F–S) for all the 27 sample subjects were analyzed using the GLM with both BDI-II and AAI as regressors. Approximately 50 voxels showed significant correlation (Z score>4.265 or P<0.00001) for any contrast and any regressor. These voxels defined the ROI that was applied to the three contrast images. Therefore, the input data consisted of 150 voxels total (50 voxels×3 contrasts).

**Figure 1:**
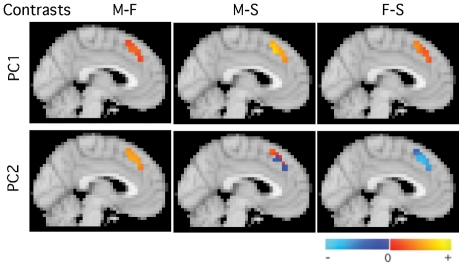
Two PC Solution. The two-PC solution across three contrast images. The ROI consists of a single cluster in the left anterior paracingulate gyrus.

Second, two principal components (PCs) were extracted from the ROI (Fig. 1). The fMRI activity in the ROI for each subject could thus be approximated as a linear combination of the 2 PCs.

The next step in the PCR approach is the multiple linear regression (MLR) between the two PCs and the psychometric data. First, MLR was used to determine the contribution of each PC to the brain activity in the ROI; this produces a coefficient for each PC.

The implementation of MLR is then straightforward linear algebra:

For the 27 sample subjects, the *SamplefMRIWeights* matrix has 3 columns – the first two columns are the coefficients for the two PCs and the last column is the constant 1, i.e. the intercept term, and 27 rows – one for each sample subject. The *SamplePsychometrics* matrix has 2 columns – one for BDI-II and one for AAI, and 27 rows – one for each sample subject. This gives us the following equation:
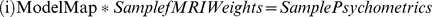
Solving for ModelMap we obtain:
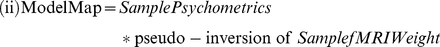
Similarly, for the test subject, the *TestfMRIWeights* matrix is a 3-column by 1-row matrix (consisting of the two PC coefficients and the intercept term), and the *PredictedPsychometrics* matrix is an equivalent 2-column by 1-row matrix (consisting of the predicted BDI-II and AAI scores). Thus, we can derive *PredictedPsychometrics* using equation (iii):
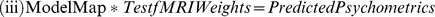
where * is the matrix multiplication and the pseudo-inversion is the Moore-Penrose pseudoinverse algorithm provided by *Matlab (Mathworks Inc. Ma.)*.

Categorical predicted diagnoses of depression were made using a predicted score threshold of >13.5 (based on the established clinical threshold score of > = 14 on the BDI-II for diagnosis of depression, and modified to deal with continuous predicted values). Categorical predicted diagnoses of insecure attachment were made using a predicted score threshold of <4 (based on Main and Goldwyn's scoring system). [26]

## Results

### 3.0 Subject Characteristics

Subject demographic data are summarized in Table 1. There were no significant between-group differences. Control Subjects BDI-II scores ranged from 0 to 12. Depressed subjects' BDI-II scores ranged from 11 to 54. Three had mild depression, including one subject depressed by MINI but with BDI-II score of 11, which is within normal range. Four had moderate depression (BDI-II 20–28), and 7 had severe depression (BDI-II 29–63). Five depressed subjects received AAI coherence of mind scores of 6 or greater (secure) and four had scores of 3 or less (insecure), with six in the indeterminate range. Of the control subjects, 9 received AAI scores of 6 or greater (secure) and 5 had scores of 4–5 (indeterminate). None had scores of 3 or less (insecure). Finally, depressed subjects showed a trend towards higher salience and valence ratings for Friend and lower salience and valence ratings for Mother and Strangers compared to control subjects. Both depressed and control subjects rated Mother and Friend higher than Strangers.

**Table 1:** Socio-Demographic and Behavioral Characteristics.

	Control Group (n = 14)		Depressed Group (n = 14)	
	*Mean*	*SD*	*Mean*	*SD*
Age (years)	24.50	2.47	24.85	3.10
Years of Education	16.79	1.89	16.63	2.12
% of days having contact with Mother^1^	53	38	46	40
% of days having contact with Friend^1^	55	36	50	43

Race/Ethnicity	*N*	*%*	*N*	*%*
Caucasian	10	71.43	11	78.57
African American	1	7.14	0	0.00
Hispanic/Latino	1	7.14	2	14.29
Asian	2	14.29	0	0.00
Other^2^	0	0.00	1	7.14

1 *% of the 365 days of the past year on which subject spoke with or saw Mother or Friend.*

2 *Pacific Islander, Alaskan Native or Native American.*

### 3.1 Diagnostic Performance

**Figure 2:**
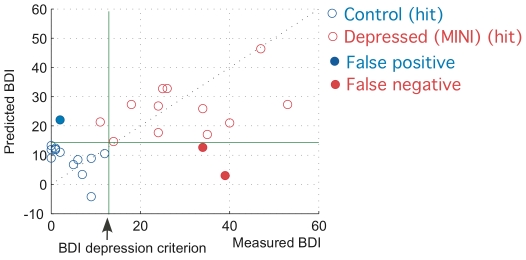
Model Performance. The predicted BDI-II (Y-axis) vs. the measured BDI-II (X-axis). Coefficient of correlation between predicted and measured BDI-II scores was r = 0.55.

The categorical agreement between the derived BDI-II score and depression diagnosis by MINI psychiatric interview was 89%, with sensitivity 85.7% and specificity 92.8%. Predicted and measured BDI-II scores had a correlation of 0.55. Our model made one false positive and two false negative diagnoses, and correctly diagnosed one subject as depressed who scored below threshold on her actual BDI-II. These results are summarized in Fig. 2, which shows the relationship between the predicted BDI-II score and the measured BDI-II scores for all subjects.

Although the depression could be diagnosed with significant accuracy from the fMRI data, insecure versus secure attachment could not be determined (the p-value for the agreement between the predicted and the measured AAI classification was non-significant at 0.16).

### 3.2 ROI and Principal Component Interpretation

**Figure 3:**
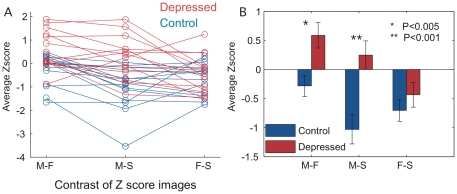
Pattern of Activity in the ROI. 3a. The average Z score for the ROI in the anterior paracingulate gyrus for each control (blue) and depressed (red) subject. 3b. Group mean average Z score for the ROI in the anterior paracingulate gyrus for control (blue) and depressed (red) subjects. Error bars represent standard error of measurement. Two-tailed p-values for group mean t-test are p<0.005 for M–F, and p<0.001 for M–S. F–S was not significant.

Approximately 50 voxels, all within a single cluster in Brodmann area 32 – the left anterior paracingulate gyrus (aPCG), showed significant correlation (Z score>4.265 or P<0.00001) with any regressor for any contrast. Activity in the ROI was significantly higher for depressed compared to control subjects for contrasts involving Mother (early/primary attachment figure). Depressed subjects showed higher activation for Mother than Friend or Stranger and control subjects showing lower activation. This suggests that mother images had the greatest impact in differentiating depressed from control subjects. This is illustrated in Fig. 3, which shows the average BOLD signal within the ROI (the colored region in Fig. 1) for the depressed and control subjects over the three contrasts. Furthermore, the pattern of relative BOLD signal across the 3 contrasts differs between control and depressed subjects. While control subjects show lowest activity for M–S among the three contrasts, depressed patients show higher M–S activity than F–S activity. Note that, although there are significant differences in mean Z scores for both M–F (p<0.005) and M–S (p<0.001) contrast between the depressed and control groups, there is also substantial overlap (Fig. 3a). Thus, ROI activity in any one contrast has poor sensitivity and specificity for the diagnosis of depression.

**Figure 4:**
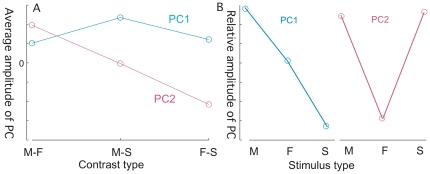
Pattern of Activity for the Principal Components. 4a. The average values within the ROI for the two major PC images. 4b. Relative values of PC derived from Fig. 4a, showing the relationship between the fMRI activity and the stimulus type. Only the pattern across stimulus types with in each PC is relevant.

The first two principal components of activity in the ROI (PC1 and PC2) provided the model with greatest diagnostic accuracy, and accounted for 22% and 13% of the variance in the ROI signal, respectively. We found that activity across the 3 viewing conditions differed for PC1 and PC2. This is can be seen on inspection of figure 1, and is illustrated quantitatively in Fig. 4a, which shows average value of the PCs for the three contrasts. The implicit relative activities for the original viewing conditions can then be derived from the contrasts. Fig. 4b represents the data in terms relative values of PC for the three original conditions – viewing Mother, Friend, and Strangers, respectively. Here we see that PC1 activity declines with level of attachment from Mother (primary/early attachment figure) to Friend (secondary/late attachment figure) to Stranger (no attachment). PC2 activity, on the other hand, is similar for Mother and Stranger and lowest for Friend, indicating it relates to some other factor.

**Figure 5:**
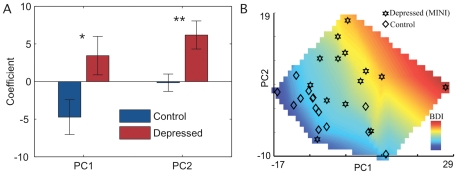
Principal Component Contributions for Depressed and Control Subjects. 5a. The coefficients for each PC. Colored bars and error bars indicate the mean and standard error, respectively, among the group. Two-tailed p-value for t-test of group-wise difference of means *p<0.05, **p<0.01. 5b. The coefficients for the two PCs for all the subjects and their BDI-II scores. The units of these coefficients are arbitrary and only their relative values are meaningful.

The PC activities also differed by diagnostic group. Depressed subjects had significantly higher levels of both PC1 and PC2 activity than control subjects (Fig. 5a). Fig. 5b shows the relationship between the coefficients of the two PCs (the values in the *SamplefMRIWeights* matrix) and the BDI-II scores. Note that, the classification line is not along any cardinal axis, indicating that each PC alone is not sufficient for predicting BDI-II and suggesting that depression involves multiple factors.

## Discussion

### 4.0 Context and Interpretations

To our knowledge this is the first study to attempt diagnosis of depression using a personalized attachment-based fMRI paradigm. Using response to viewing Mother and others, we found depression could be diagnosed with a model based on activity patterns in the Anterior Paracingulate Gyrus (aPCG, Brodmann Area 32). It is notable that while our model predicted depression robustly, it was not able to predict attachment security. The attachment system is activated under conditions of threat or distress, making attachment figures more salient [29]. Thus, in depressed subjects, the incentive salience of attachment figures such as Mother may associate with characteristic brain activity patterns [30]. However, it is possible that the inter-subject variability for the AAI is too large for a satisfactory prediction of attachment security itself, independent of depression. Further, the neurobiology of attachment security may be more complex than that of depression. Thus, while the first two principal components do not provide sufficient information to diagnose attachment security, the signal to noise ratios of other minor PCs are not strong enough to improve prediction. The correlation between predicted and actual AAI scores was moderate, 0.31, suggesting that a larger sample population with greater power might also allow prediction of AAI. However, it is also possible that the complexity of attachment security derives from significant non-linearity in the activity signature of its neural substrate and/or greater variability or error in its clinical measurement.

In addition to our hypothesis that a personalized attachment-based paradigm could provide robust diagnosis of depression, we hypothesized that using a linear multivariate model with an approach that favored signal-to-noise ratio over model performance as a feature selection criterion would provide clinically interpretable results. It is notable that although there were almost no significant behavioral differences between depressed and control subjects during scanning, brain activity patterns differed significantly.

In our model, we found that higher levels of aPCG activity associated with depression, however only the pattern of aPCG activity was sensitive and specific to the diagnosis. The aPCG is involved in regulating affect and in representing affects of self and others [31], [32], [33], [34]. This area has also been implicated in error and conflict resolution [35] and as a regulator with functional connections to other frontal areas involved in social appraisal [36]. Oxytocinergic activity in the hypothalamus has been linked in studies of attachment to reward processing activity in the ventral striatum [37] and is in turn regulated by the aPCG and other areas of the medial PFC [17]. Higher activity in this area for depressed subjects during appraisal of attachment figures and others could reflect compensatory control activity in a dysregulated network, as has been suggested in various studies [38], [39], [40]. This activity might serve to resolve conflict experienced in the appraisals subjects made [41] to compensate for decreased regulatory function in the subgenual cingulate [42]. Indeed, depressed subjects exhibited lower variability in their ratings for all viewing conditions than did controls. Moreover, decreases in resting metabolism in this area were observed to correlate with response to Inter-Personal Therapy (IPT) treatment for depression [8].

Though higher overall levels of activity in the aPCG associated significantly with depression, they were not diagnostic. Rather the pattern of activity was. This pattern consisted of two principal components. The first and dominant PC appeared to be a function of degree of attachment, while the second PC appeared to relate to an attachment-independent process.

**Figure 6:**
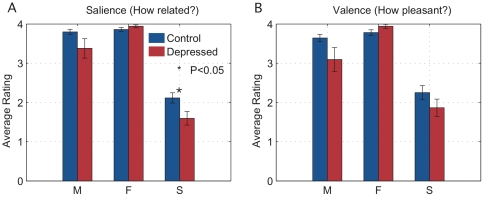
Subject Ratings of Stimuli. 6a. The salience scores (average across trial for each subject), on a 1–4 scale, for the three viewing conditions and both groups of subjects. 6b. The valence scores, on a 1–4 scale, for the three viewing conditions and both groups of subjects.

Depression was diagnosed when the sum of the principal component loadings was above a threshold value. Thus, either pattern, if sufficiently strong, or the combination, if more moderate, predicted depression. The first and dominant pattern, described by PC1, is of increasing activation intensity as the degree of attachment between subject and stimulus increases (Fig. 4b). This activity was heighted in depressed subjects and suppressed in controls (Fig. 5a). A comparison with subject salience ratings recorded during viewing (Figure 6a) shows no significant difference between depressed subjects and controls in their ratings of Mother and Friend. Depressed subjects compared to controls also showed decreased variability in their responses in different viewings of the same stimulus. Thus the behavior of PC1 may reflect regulatory overcompensation in response to emotions evoked by attachment figures, and in particular, Mother.

The activity pattern described by PC2 (Fig. 4b) on the other hand, which is low for Friend and high for both Mother and Stranger, may reflect heightened regulation in response to an interpersonal process independent of and distinct from attachment. It is conceivable, for example, given the very positive ratings (with near-zero intra-subject variability) given to Friends by depressed subjects (Fig. 6), that this activity relates to ambivalence towards Mother and Strangers experienced by depressed subjects. Indeed, depressed subjects tended to rate Mother and Stranger more negatively than did the control subjects, with trend towards significance. (Fig. 6)

### 4.1 Limitations

Several important limitations should be considered. While fMRI provides an objective measure of depression, the major limit of this method is that it is still not sensitive enough to provide diagnosis of mild depression. However, it is important to emphasize that we did not expect to find a perfect agreement between the derived and measured BDI-II for a variety of reasons. Both fMRI and BDI-II measures are subject to measurement errors and individual differences that are unique for different measures. Since the sources of error in fMRI measures and psychometrics such as the BDI-II should be independent, we should be able to combine probabilities derived from each to increase the sensitivity/specificity of a combined test for depression. Ultimately, however, the clinical utility of diagnostic fMRI will likely lie in differentiating disorders which are not reliably clinically distinguishable on cross-sectional examination, such as unipolar and bipolar depression [12]. This study, though, is not designed to test such an application.

Although attachment is a major factor for depression, other behaviorally symptomatic features, such as reward- and loss-discounting can contribute to diagnostic power. [9] Though our findings support the importance of attachment related measures in the assessment of depression they do not provide a comparison between the power of this measure and others. Similarly, though our finding that ROI activity differed significantly between depressed and controls only for contrasts with Mother suggests that heightened response to Mother is the key to diagnosis, it remains possible that parallel lowered response to Friend and Stranger stimuli is the deciding factor. Further, we cannot rule out that familiarity rather than attachment accounts for the activity differences between Mother and Stranger. However, the significant but non-diagnostic correlation between activity in the aPCG and AAI score seen in all three contrasts suggests that the activity is related to attachment status rather than mere familiarity.

Another possibility is that the effect of Mother images is a result of administration of the AAI on the morning of the scan, possibly reactivating childhood and emotional memories which would not oterwise have been elicited by viewing one's mother's image. Thus, while it remains clear that the attachment-based paradigm allowed for diagnosis of depression, it is not clear if the AAI should be considered a part of this paradigm or not.

It should be noted that our PCR method may involve a trade-off between classificatory power and interpretability: We do not use all the data that could have some valuable information (classificatory power disadvantage compared to SVC and PLS). We do not consider nonlinear relationships (classificatory power disadvantage compared to SVC). And we do not consider complex interactions (classificatory power disadvantage compared to SVC). Finally, while the leave-one-out approach ensures that test data are independent of the sample data, a more definitive approach would be to use separate test and sample cohorts.

### 4.2 Conclusions

Functional magnetic resonance imaging using personalized paradigms has the potential to provide objective assessments, even when behavioral measures are not informative. Brain activity patterns in response to viewing one's mother are sensitive and specific correlates of depression. These activity patterns are dominated by a principal component that appears to be a function of degree of attachment in combination with a second principal component, which appears to relate to an attachment-independent process.

Although the BDI-II, a depression index measured with a questionnaire and the neural activity related to the viewing of attachment figures are dramatically different in nature, using the PCR approach described here in combination with a personalized, attachment-based paradigm, the BDI-II can be derived from the neural activity recorded with fMRI. The underlying connection between attachment and depression is manifested as a close to linear relationship between the fMRI data and the BDI-II.

Finally, the interpretability of our PCR model suggests that fMRI based diagnostic algorithms may enhance our understanding of the neural mechanisms of depression by identifying distinctive neural features of the illness.
